# Legibility Assessment of Visual Word Form Symbols for Visual Tests

**DOI:** 10.1038/s41598-019-39408-7

**Published:** 2019-03-04

**Authors:** Li-Ting Tsai, Yuh Jang, Kuo-Meng Liao, Chien-Chung Chen

**Affiliations:** 1 Department of Early Childhood Care and Education, University of Kang Ning, Taipei, Taiwan; 2 Taiwan Association for Visual Rehabilitation, Taipei, Taiwan; 3 School of Occupational Therapy, College of Medicine, National Taiwan University, Taipei, Taiwan; 4 Division of Endocrinology and Metabolism, Department of Internal Medicine, Zhong-Xiao branch, Taipei City Hospital, Taipei, Taiwan; 5 Department of Psychology, National Taiwan University, Taipei, Taiwan

## Abstract

For a reliable visual test, it is important to evaluate the legibility of the symbols, which depends on several factors. Previous studies have compared the legibility of Latin optotypes. This study developed a visual function test based on identification visual capacity for a Chinese reading population. The legibility of word symbols was assessed with three methods: (1) Identification of the contrast thresholds of the character sets, (2) patterns of confusion matrices obtained from analysis of the frequency of incorrect stimulus/response pairs, and (3) pixel ratios of bitmap images of Chinese characters. Then characters of similar legibility in each character set were selected. The contrast thresholds of the final five character sets and the Tumbling E and Landolt C optotypes were evaluated. No significant differences in contrast threshold were found among the five selected character sets (p > 0.05), but the contrast thresholds were significantly higher than those of the E and C optotypes. Our results indicate that combining multiple methods to include the influences of the properties of visual stimuli would be useful in investigating the legibility of visual word symbols.

## Introduction

A typical visual function test usually uses simple, standardized visual optotypes to examine the visual capacity of patients^1,2^. However, for reliable data collection and accurate diagnosis, the development of a visual test actually involves very complex procedures, from optotype selection and test construction to psychometric validation, before the test can be applied in a clinical setting^3,4^.

The Landolt C, Tumbling E, and Sloan letters, the most popular optotypes, have been used extensively and globally in several kinds of clinical vision tests. However, an important question is whether they are the most suitable optotypes for testing the functional vision capacities of people whose primary language does not use Latin letters^5^. The Sloan letter test, which requires the patients to name the letters, is obviously not suitable for patients with limited knowledge of the Latin alphabet. It is possible to use the Landolt C or Tumbling E charts for people who cannot read Latin optotypes by instructing the patients to respond to the orientation of the gap in an E or C, and quite a number of studies support the relationship between the visual acuity and functional vision performance^6,7^. However, debate continues on the nature of the mechanisms underlying the visual acuity measured with the Landolt C or Tumbling E^8,9^. It is possible that such orientation identification tasks measures only resolution acuity rather than recognition acuity^10,11^. The latter, as measured with the Sloan letters, includes more compensatory top-down cognitive components and may be more related than the former to functional vision performance^10,12^.

Whether or not Latin optotypes are used, one of the major concerns about the use of visual symbols to measure visual function is their legibility^4,13^. The legibility of visual symbols depends on the physical properties of the symbols, such as size, contrast, font type, and spacing^13,14^. Thus, one classical approach to assess the legibility of visual symbols, as used in the development of the Sloan letter chart^1,13,15,16^, is to compare the percentage of correct responses for each optotype in an optotype set and to conduct an error analysis of the incorrect responses at the limit of visual acuity or contrast threshold. The other popular way to estimate the legibility is to use psychophysical methods to compare the size thresholds or contrast thresholds of the individual symbols^5,17,18^. Other techniques, such as Fourier frequency spectrum analysis^19^, stroke frequency^5,17^ and the image descriptors method^20^, have also been proposed for assessing legibility.

Previous studies often used the size threshold of a high contrast target for assessing legibility^1,5,13,17^. However, contrast is also an important criterion for legibility, which can vary under high or low contrast conditions for the same alphabetic characters or visual symbols^16,18,21–23^. The Sloan letters were originally chosen to have similar legibility for visual acuity assessment^1^, but these letters are also used for contrast sensitivity (e.g., the Pelli-Robson chart)^21^. A fixed size and various contrasts of letters are used for the Pelli-Robson chart, and Robson *et al*. found only smaller differences between an acuity chart and a contrast chart^22^. However, Elliot, Whitaker, and Bonette reported that the legibility of Sloan letters assessed at the contrast threshold was different from that assessed at the acuity threshold^16^. Alexander *et al*. reported that the Sloan letter optotypes showed relatively greater interletter variability of threshold log contrast for small letters than for large letters or threshold log MAR for high-contrast letters^18^. These results show the importance of contrast in optotype legibility. Therefore, it is necessary to consider the performance near the contrast threshold when assessing legibility.

To the best of our knowledge, there is no standardized equivalent test to measure acuity with traditional Chinese characters. The purpose of this study was first to develop a method to investigate the legibility of Chinese characters, a type of visual word symbol, and then to group visual word symbols of similar legibility so as to develop a visual function test that assesses identification visual capacity for a Chinese reading population. To this end, based on the properties of symbols, multiple methods (identification contrast threshold, patterns of confusion matrices, and pixel ratio) were used to estimate the legibility of Chinese characters presented to the fovea. For comparison with current established tests, we also compared the legibility of Chinese characters with those of the E and C optotypes to examine whether there was a significant difference in legibility between Chinese characters with a simple structure and Latin optotypes. We used contrast threshold, instead of size threshold, as a critical criterion to determine the legibility. The rationale behind this choice was the importance of contrast threshold in legibility assessment, as discussed above^16^, and the need for clinical application in the assessment of contrast-related functional vision^24,25^.

## Results

### Character selection

**Figure 1:**
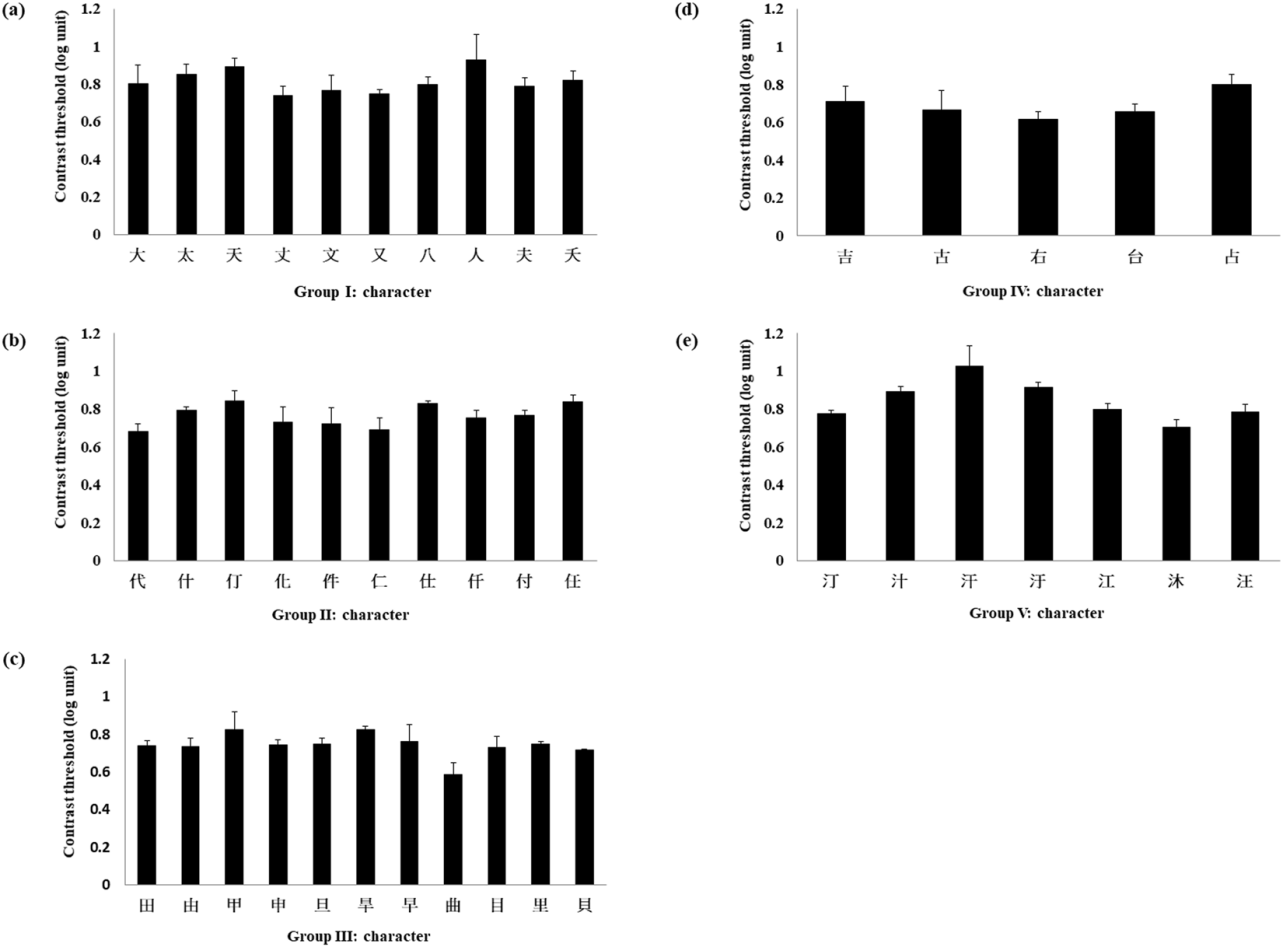
Identification contrast thresholds for the original five character group sets (from panel (a) to (e): stage one, character selection). The contrast threshold of each character was the average of the contrast at the last five reversals in all 4 repetitions of the same condition. Means of contrast threshold were mostly located between 0.7 log unit and 0.9 log unit. The error bars represent 1 standard deviation (SD).

The legibility of a character was evaluated with contrast threshold, patterns of confusion matrices, and pixel ratio. The contrast threshold of each character (Fig. 1) was the average of the contrast at the last five reversals in all 4 repetitions of the same condition for each participant. The mean of the identification contrast threshold for all 43 characters was 0.77 ± 0.08 log unit. The average values of the contrast thresholds of the five character sets were 0.81 ± 0.06 log unit (Group I), 0.76 ± 0.06 log unit (Group II), 0.74 ± 0.06 log unit (Group III), 0.68 ± 0.07 log unit (Group IV), and 0.84 ± 0.11 log unit (Group V), respectively. The error bars represent 1 standard deviation (SD). Characters with relatively larger variation in contrast threshold among the participants were excluded from the final test. The pixel ratio, indicating stroke density, as discussed above, was the number of the total pixels composing the strokes divided by the number of pixels in the whole character image (Table 1).

**Table 1:** Chinese characters, corresponding configurations of character, and pixel ratios of character image.

A		B		C		D		E	
Configurations									
single		left-right		surrounding		top-down		left-right	
Symbol	Ratio	Symbol	Ratio	Symbol	Ratio	Symbol	Ratio	Symbol	Ratio
**大da**	18.8	代dai^*^	24.0	田tian^*^	27.8	**吉ji**	24.7	汀ting^*^	18.0
**太tai**	20.6	什she^*^	20.2	由you^*^	27.1	**古gu**	22.0	汁zhi^*^	17.7
**天tian**	21.9	仃ding^*^	20.2	甲jia^*^	24.9	右you^*^	23.6	汗han^*^	20.7
丈jang^*^	19.8	化hua^*^	22.3	申shen^*^	24.4	台tai^*^	23.7	**汙wu**	21.0
文wen^*^	21.0	件jian^*^	26.2	**旦dan**	23.5	**占zhan**	20.5	**江jiang**	20.3
又you^*^	19.0	仁ren^*^	17.2	旱han^*^	27.4	—		沐mu^*^	25.6
八ba^*^	14.0	**仕shi**	24.0	早zao^*^	25.6	—		**汪wang**	23.5
人ren^*^	13.8	**仟qian**	23.2	曲qu^*^	33.0	—		—	
夫fu^*^	23.0	付fu^*^	24.1	**目mu**	24.2	—		—	
**夭yao**	21.0	**任ren**	26.6	里li^*^	32.0	—		—	
—		—		**貝bei**	26.9	—		—	

Note: Characters in each set (A to E) were similar in structure and configuration. Pixel ratios, contrast threshold, and patterns of confusion matrices were used in legibility assessment. Only characters without any mark and in bold were selected.

^*^Characters excluded based on the criteria of character selection.

Tables 2–6 shows the confusion matrices averaged across participants. The confusion matrix was computed by counting the types of the incorrect response in the last five reversals of each run. Each column represents a stimulus presented to the participants. The rows show the probability of each character being selected by the participants. Cells, which are the probability of selecting a particular character given a stimulus, having a confusion rate exceeding 15% are underlined because they indicate character pairs with a greater possibility that participants may confuse them. Highly confusable characters are more valuable for testing the limits of contrast vision than are ones that are easily distinguished, so these character pairs were considered good confusion pairs.

**Table 2:** Confusion matrices of the Group A character sets (first stage) Stimulus.

		Stimulus									
		大	太	天	丈	文	又	八	人	夫	夭
**Incorrect response (%)**	**大**		60.0	30.0	8.3	6.7	6.7	13.3	56.7	28.3	18.3
	**太**	16.7		0.0	6.7	3.3	3.3	16.7	3.3	0.0	6.7
	**天**	36.7	15.0		8.3	6.7	5.0	13.3	21.7	35.0	36.7
	**丈**	3.3	3.3	1.7		26.7	13.3	8.3	0.0	13.3	5.0
	**文**	1.7	5.0	0.0	40.0		41.7	5.0	0.0	5.0	8.3
	**又**	1.7	1.7	3.3	10.0	43.3		15.0	1.7	5.0	6.7
	**八**	3.3	3.3	1.7	5.0	1.7	1.7		6.7	0.0	5.0
	**人**	20.0	3.3	3.3	10.0	5.0	21.7	16.7		10.0	5.0
	**夫**	11.7	1.7	20.0	8.3	1.7	3.3	5.0	5.0		8.3
	**夭**	5.0	6.7	40.0	3.3	5.0	3.3	6.7	5.0	3.3	

Note: Percentages over 15% were underlined, and were defined as good pairs of confusion, indicating that the paired characters had higher percentages of confusion with each other near the identification contrast threshold. When a character formed a good confusion pair with more characters, it had a greater chance of being included in experiment 2. X-axis, stimuli; Y-axis, incorrect responses in percentage frequency (%).

**Table 3:** Confusion matrices of the Group B character sets (first stage) Stimulus.

		Stimulus									
		代	什	仃	化	件	仁	仕	仟	付	任
**Incorrect response (%)**	**代**		3.3	0.0	11.7	10.0	15.0	3.3	1.7	5.0	0.0
	**什**	18.3		20.0	21.7	26.7	16.7	33.3	31.7	26.7	3.3
	**仃**	6.7	11.7		8.3	6.7	10.0	1.7	15.0	21.7	5.0
	**化**	18.3	6.7	1.7		3.3	0.0	15.0	8.3	5.0	5.0
	**件**	13.3	10.0	6.7	5.0		3.3	1.7	8.3	13.3	5.0
	**仁**	13.3	5.0	1.7	16.7	3.3		6.7	8.3	10.0	6.7
	**仕**	11.7	15.0	6.7	16.7	10.0	33.3		5.0	5.0	45.0
	**仟**	6.7	30.0	20.0	3.3	23.3	1.7	3.3		10.0	26.7
	**付**	6.7	11.7	20.0	8.3	5.0	10.0	3.3	3.3		3.3
	**任**	5.0	6.7	23.3	8.3	11.7	10.0	31.7	18.3	3.3	

Note: Percentages over 15% were underlined. X-axis, stimuli; Y-axis, incorrect responses in percentage frequency (%).

**Table 4:** Confusion matrices of the Group C character sets (first stage) Stimulus.

		Stimulus										
		田	由	甲	申	旦	旱	早	曲	目	里	貝
**Incorrect response (%)**	**田**		26.7	5.0	3.3	5.0	0.0	3.3	11.7	5.0	10.0	5.0
	**由**	31.7		13.3	18.3	3.3	3.3	10.0	21.7	3.3	3.3	3.3
	**甲**	1.7	10.0		40.0	3.3	1.7	8.3	6.7	0.0	11.7	1.7
	**申**	5.0	11.7	36.7		3.3	5.0	5.0	8.3	5.0	10.0	6.7
	**旦**	8.3	5.0	0.0	3.3		0.0	8.3	6.7	36.3	16.7	26.7
	**旱**	1.7	0.0	1.7	0.0	10.0		33.3	6.7	3.3	8.3	0.0
	**早**	1.7	1.7	16.7	0.0	10.0	70.0		15.0	5.0	10.0	3.3
	**曲**	18.3	28.3	3.3	10.0	3.3	5.0	10.0		18.3	10.0	5.0
	**目**	10.0	6.7	3.3	3.3	33.3	1.7	11.7	18.3		11.7	40.0
	**里**	15.0	1.7	20.0	20.0	11.3	10.0	5.0	3.3	3.3		8.3
	**貝**	6.7	8.3	0.0	1.7	17.0	0.0	5.0	1.7	20.3	8.3	

Note: Percentages over 15% were underlined. X-axis, stimuli; Y-axis, incorrect responses in percentage frequency (%).

**Table 5:** Confusion matrices of the Group D character sets (first stage) Stimulus.

		Stimulus				
		吉	古	右	台	占
**Incorrect response (%)**	**吉**		25.0	11.7	17.0	5.0
	**古**	63.3		30.0	21.7	83.3
	**右**	20.0	38.3		48.0	3.3
	**台**	8.3	18.3	41.7		8.3
	**占**	8.3	18.3	16.7	13.3	

Note: Percentages over 15% were underlined. X-axis, stimuli; Y-axis, incorrect responses in percentage frequency (%).

**Table 6:** Confusion matrices of the Group E character sets (first stage) Stimulus.

		Stimulus						
		汀	汁	汗	汙	江	沐	汪
**Incorrect response (%)**	**汀**		18.3	13.3	28.3	8.3	6.7	10.0
	**汁**	13.3		18.3	13.3	15.0	34.7	10.0
	**汗**	11.7	48.3		41.7	3.3	13.0	21.7
	**汙**	31.7	11.7	60.0		15.0	20.0	10.0
	**江**	31.7	11.7	0.0	3.3		6.0	48.3
	**沐**	5.0	6.7	1.7	3.3	5.0		0.0
	**汪**	6.7	3.3	6.7	10.0	53.3	19.7	

Note: Percentages over 15% were underlined. X-axis, stimuli; Y-axis, incorrect responses in percentage frequency (%).

Based on these results, we were able to select the characters for the final test. We applied three criteria: (1) Good pairs of characters should produce more than 15% error in the confusion matrix. Thus, characters that produced confusion of less than 15% were excluded. (2) Characters with contrast thresholds deviating from the mean by more than 1.5 standard deviation (SD) were excluded to ensure the homogeneity of performance for the whole data set. (3) Finally, we kept only three to four characters in each dataset that have the most similar pixel ratios. The excluded characters are marked by asterisks in Table 1. Finally, 16 characters (Table 1 with bold marks) were selected from the original five character sets composed of 43 characters.

### Comparison with alphabetic optotypes

**Figure 2:**
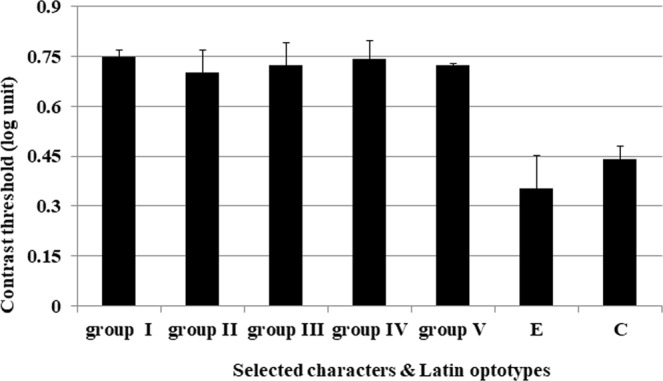
Identification contrast threshold and standard deviation for the final character sets, Tumbling E, and Landolt C optotypes.We demonstrated the identification contrast threshold according to their original character sets. Significant difference was observed between character sets and Latin optotypes (P < 0.05, t-test). (n = 8).

We then compared the contrast threshold of the characters in our final test set with those of the Tumbling E and Landolt C optotypes. Figure 2 shows the contrast thresholds averaged across characters in each final character set and those of the Tumbling E and Landolt C optotypes. The average values of the contrast threshold of the five new selected character sets were 0.73 ± 0.02 log unit. Mean contrast thresholds for individual character sets, which ranged from 0.70 (for group II) to 0.75 (for group I) log unit, were similar among the Chinese character sets. The identification contrast thresholds of the Latin optotypes were 0.35 (SD = 0.10) and 0.44 (SD = 0.04) log units for the Tumbling E and Landolt C, respectively. We used the independent t test for comparing the mean contrast thresholds between different groups. No significant differences in contrast threshold were found among the five selected character sets (p > 0.05). However, independent t-test suggested significant differences between our character sets and the Latin optotypes (p < 0.05).

## Discussion

The main focus of this study was to select characters for the development of a visual test for the Chinese reading community by measuring the legibility of traditional Chinese characters. We applied the identification contrast threshold, confusion matrix and pixel ratio to identify a set of characters suitable for a clinical test. Sixteen characters were selected for the test from 43 assessed characters^26^.

We also compared the legibility of the 16 traditional Chinese characters to those of the Landolt C/tumbling E optotypes, which are used in most clinical eye examinations. The results showed that the contrast threshold of Chinese characters, even those with simple structures, was significantly higher than those of the Tumbling E (2.3%) and Landolt C (2.8%) optotypes. A possible reason for the effect is the differences in the spatial complexities of our stimuli and the E or C optotypes. Pelli *et al*. measured the identification efficiency for various types of visual word forms and checkerboard patterns^27^. They found that efficiency decreases with spatial complexity; in particular, the efficiency of identification is lower for Chinese characters than for Latin letters^27^. They argued that the difference in efficiency results from the likelihood to form “goodness” (in Gestalt theories of visual perception) in characters. Our result is consistent with theirs and thus may reflect the same difference in visual processing of complex characters and simple letters.

We did not directly compare the legibility of our characters and that of the Sloan letters, for the latter are not generally used in clinical settings in Taiwan, where this study was performed. Nevertheless, a previous study has compared them^5^. Zhang *et al*. showed that an observer could identify a Sloan letter at a smaller size than they could the simplest Chinese characters (e.g., 山, 人, 力) with a smaller stroke frequency^5^. Our characters had higher spatial complexity than the easiest character group in the study by Zhang, *et al*.^5^, so the legibility of our characters could be lower than that of the Sloan letters.

Thus, the legibility of our characters was lower than those of the Landolt C and Tumbling E and is very likely to be lower than that of the Sloan letters. This is not surprising, for our characters are spatially more complex than those of the Latin optotypes. The participants may have needed to expend more effort to identify these characters than to identify the Latin optotypes. It should be noted that the main purpose of this test was not to replace the Landolt C and Tumbling E, which are good tools for assessing visual acuity, but to assess a patient’s functional vision, which highly depends on the cultural background of the patient. Thus, we believe that the test we developed here should be more relevant than Latin optotypes to the functional vision of a patient in Taiwan. In addition, our methods to determine the legibility could be adopted for other countries that their primary language does not use Latin letters.

The use of several configurations of similar Chinese characters was modelled after the Sloan letter set^18^, which contains various types of strokes and subgroups of letter sets, such as C, D, and O, which tend to be confused at the acuity or contrast threshold. Our study also showed similar results in the confusion matrix of the final character set in the experiment of comparison with alphabetic optotypes (Table 7). The results showed that the characters formed close relations of confusion only near the contrast threshold with characters from their original character set (marked with underline).

**Table 7:** Confusion matrix of the final character set Stimulus.

		Stimulus															
		大	太	天	夭	仕	仟	任	旦	目	貝	吉	古	占	汙	江	汪
**Incorrect response (%)**	**大**		*66.7*	*22.2*	8.9	2.2	0.0	2.2	2.2	2.2	0.0	2.2	0.0	4.4	0.0	0.0	0.0
	**太**	*22.2*		2.2	8.9	2.2	0.0	4.4	0.0	0.0	0.0	2.2	0.0	2.2	0.0	0.0	0.0
	**天**	*33.3*	11.1		*62.2*	2.2	0.0	0.0	2.2	0.0	6.7	2.2	0.0	4.4	0.0	4.4	0.0
	**夭**	17.8	4.4	*40.0*		2.2	4.4	0.0	0.0	0.0	2.2	0.0	0.0	2.2	4.4	2.2	0.0
	**仕**	2.2	2.2	11.1	2.2		17.8	28.9	6.7	8.9	2.2	4.4	4.4	4.4	2.2	2.2	4.4
	**仟**	8.9	4.4	6.7	2.2	*20.0*		17.8	6.7	15.6	6.7	0.0	4.4	4.4	11.1	2.2	4.4
	**任**	0.0	4.4	2.2	2.2	*48.9*	*57.8*		8.9	0.0	0.0	2.2	4.4	2.2	11.1	0.0	2.2
	**旦**	0.0	0.0	0.0	2.2	0.0	0.0	2.2		*33.3*	8.9	2.2	4.4	6.7	0.0	4.4	2.2
	**目**	4.4	0.0	2.2	2.2	2.2	6.7	8.9	*33.3*		*60.0*	13.3	17.8	2.2	2.2	6.7	4.4
	**貝**	2.2	0.0	0.0	0.0	0.0	2.2	4.4	13.3	*26.7*		2.2	6.7	0.0	6.7	4.4	0.0
	**吉**	2.2	0.0	2.2	0.0	2.2	0.0	0.0	0.0	2.2	2.2		*20.0*	6.7	4.4	2.2	0.0
	**古**	0.0	0.0	0.0	4.4	0.0	0.0	8.9	6.7	6.7	4.4	*55.6*		*48.9*	8.9	2.2	2.2
	**占**	0.0	0.0	2.2	0.0	4.4	2.2	0.0	2.2	2.2	2.2	6.7	*28.9*		2.2	4.4	4.4
	**汙**	2.2	6.7	2.2	2.2	4.4	4.4	6.7	4.4	2.2	0.0	4.4	2.2	2.2		11.1	*31.1*
	**江**	2.2	0.0	4.4	0.0	8.9	2.2	4.4	8.9	0.0	2.2	0.0	4.4	2.2	*20.0*		*44.4*
	**汪**	2.2	0.0	2.2	2.2	0.0	2.2	11.1	4.4	0.0	2.2	2.2	2.2	6.7	*26.7*	*53.3*	

Note: We analyzed the confusion matrix of the experiment of comparison with alphabetic optotypes. We found that the characters only formed close relations of confusion with characters from their original character set. Column: stimuli; Row: corresponding incorrect response in percentage frequency (%).

The results on these 16 Chinese characters of similar legibility have been applied to a new macular visual function test, named the Macular Multi-Function Assessment (MMFA)^26^. In that study, Chinese characters were adopted by the MMFA in the forms of 4 contrast levels (80%, 25%, 10%, and 5% contrast) to assess macular visual function in patients with Type II diabetes and in controls^26,28^. The results showed that the scores of the MMFA and Early Treatment Diabetic Retinopathy Study (ETDRS) contrast acuity charts displayed high levels of agreement and similar discriminative ability^28^. In addition, most of the MMFA scores showed significant differences between the diabetic group and the control group^26^. Therefore, the results support that the methods used in our study to examine the legibility of Chinese characters would be useful and reliable for clinical application in the use of visual word form symbols as the optotypes instead of the Landolt C or Tumbling E.

Unlike the generality of the Landolt C, tumbling E, or even the Sloan letters, which are relatively simple and have been extensively studied for decades^1,4,29,30^, the generality of our findings for different ophthalmological or neurological diseases has yet to be tested. It is possible that patients with different diseases would yield different response patterns to our character set. After all, different diseases may produce impairment of different visual functions. Further studies are needed to investigate the validity of our test in characterizing character identification performance in various visual disorders.

## Conclusions

Traditional Chinese characters have high spatial complexity, so it is not easy to use a single method to examine and present the legibility of Chinese characters. We applied multiple criteria based on identification contrast thresholds, analysis of patterns of confusion matrices, and comparisons of pixel ratios to examine the legibility of logographic visual word symbols. Based on this work, we were able to construct a test for the visual assessment of persons with Chinese as their first or only language.

## Methods

### Participants

Eight participants (5 female, 3 male, mean ages 29.8 ± 5.6 years) participated in this experiment. All participants were recruited from the campus of National Taiwan University and had normal or corrected-to-normal visual acuity (20/20). All of the procedures were reviewed and approved by the Institutional Review Board of the Taipei City Hospital, and all tests were conducted in accordance with the tenets of the Declaration of Helsinki. Informed consent was obtained from each participant when they understood the procedures of this study.

### Inclusion of characters

Given the sheer number of Chinese characters and their wide range of spatial complexity, it was impractical to include all Chinese characters in this study. Thus, some principles for characters selection were developed.

The spatial complexity of a Chinese character basically depends on (1) stroke number and (2) configuration^17,31,32^. Strokes are the basic features of a character, such as the dots, lines, slants, and hooks, of a character. The configuration is the spatial relation between character components, such as left-right (e.g., 江, /jiang/), surrounding (e.g., 田, /tian/), or top-down (e.g., 古, /gu/)^17^. Configuration is important for classifying the spatial compositions of Chinese characters, as a skilled Chinese reader often cannot identify a character when its components are in the wrong configuration and is more likely to confuse characters of the same configuration but not those of different configurations^17,31–33^. The development of the Sloan letter chart also took configuration into consideration and thus includes letters of various forms, such as vertical, oblique, and curved contours^4^.

The characters were selected from the Report on the Survey of Characters and Words Frequently used by Elementary School Children in Taiwan^34^. That report lists 5,021 commonly used characters. Only characters with fewer than 10 strokes were included in this study. The reason for this criterion was to ensure that the difficulty level would be appropriate for most readers and thus suitable for clinical applications, as Taiwanese elementary school children in grades 1 and 2 should be able to recognize most of the high frequency characters with 1 to 10 strokes^35^. Second, only characters within a category, defined by character configuration and spatial similarity (see below), containing at least five characters were included. We used only the Ming typeface of the characters. This typeface is considered to be the most legible on VDTs and is a popular typeface form^17^.

In total, five character sets composed of 43 characters, shown in Table 1, in four configuration patterns (single, left-right, surrounding, and top-down) were chosen for this study. The number of strokes of each character ranged from 3 to 7 (mean = 4.98, SD = 1.28). The characters in every set had similar numbers of strokes, forms, and configurations. The pixel ratios, defined as the number of pixels of all strokes divided by that of the whole character image (159*159 pixels) for each individual character, are shown in Table 1.

For the purpose of investigating character legibility, the character image was scaled to a 1 × 1 degree visual angle to achieve basic comfortable visibility for all of the characters^36^. The characters were presented on a display that had a mean background luminance of 89.2 cd/m^2^ at a viewing distance of 50 cm.

### Apparatus

Stimuli were displayed on a ViewSonic monitor (G90fB 19″) driven by a MacBook Pro with an Intel HD Graphics 3000 display card. The stimuli were generated by Psykinematix software with the Mono 10.8 bit bit-stealing method to reach 10 bits of contrast resolution^37^. The gamma correction was performed with the Psykinematix software and Eye-one Display 2 together. The monitor resolution was 1280 (H) × 1024 (V), and the refresh rate was 85 Hz.

### Protocol of legibility investigation

This protocol comprised two parts. The first stage was the investigation of Chinese character legibility by the psychophysical method. This information was then used to further screen the characters for the final character set. The second stage was the comparison of legibility between the selected characters and the E and C optotypes.

The first stage contained five conditions, one for each of the five character sets (Table 1). Each condition was repeated four times. The order of the 20 runs (5 conditions * 4 repetitions) was randomized. Every run contained 5 to 11 interleaved staircases, depending on the number of characters in each character set. The initial Weber contrast of a character was set at 80%. Participants were instructed to view the characters binocularly and to maintain their attention on the fixation mark at the center of the display. A character was then shown in the center of the display. The participants were instructed to press a corresponding button on the keyboard to indicate what character they perceived. Each character was presented for 250 ms, which was sufficiently long for the participants to identify the character^38^. Auditory feedback was provided to indicate correct or incorrect responses. Three participants participated in the first stage.

In the second stage, characters of similar legibility, according to the results of the first stage, were combined into a new character set. For comparison, we also measured the contrast threshold for the Landolt C and Tumbling E optotypes for eight participants, including the three who had participated in the first stage. The protocol of the psychophysical method of the second stage session was the same as that of the first stage session. The independent t test was used to compare the mean contrast thresholds between different groups.

Participants were given ample time to study the characters before the experiment. Practice trials were given at the beginning of the experiment to decrease the learning effect. Experiments were conducted in a dark room. Contrast thresholds were measured using interleaved multiple 3-down, 1-up staircase procedures. That is, the contrast of a test character decreased after three correct responses and increased after one wrong response. This procedure yields a threshold level of 79.4% correct responses. The contrast decrease rate was 50% before the first reversal and 12.5% after the first reversal, and the increase rate was 25%. Each run was terminated after 6 reversals. In total, there were at least 37 trials and on average 62.73 trials for each run. The standard error of the last 5 reversals, averaged across all trials, was about 0.63%, showing the stability of our measurements.
